# Changes in Tissue Oxygen Saturation in Response to Different Calf Compression Sleeves

**DOI:** 10.1155/2015/857904

**Published:** 2015-09-08

**Authors:** T. Dermont, L. Morizot, M. Bouhaddi, A. Ménétrier

**Affiliations:** ^1^EA3920 Marqueurs Pronostiques et Facteurs de Régulations des Pathologies Cardiaques et Vasculaires, Plateforme Exercice Performance Santé Innovation, SFR FED 4234, Université de Franche-Comté, Besançon, France; ^2^Université Savoie Mont Blanc, Chambéry, France; ^3^Physiologie-Exploration Fonctionnelles, CHRU de Besançon, France

## Abstract

*Aim*. The purpose was to examine the changes in tissue oxygen saturation (StO_2_) in response to the application of different commercially available calf compression sleeves. *Methods*. Eight subjects came to the laboratory to complete a session in seated position including 10 min of quiet rest followed by 3 min measuring calf StO_2_ without compression sleeves and then alternating of 3 min of passive rest and 3 min measuring StO_2_ with calf compression sleeves. A total of 15 different commercially available compression sleeves were studied in a randomized order. Calf StO_2_ was recorded using near-infrared spectroscopy. *Results*. StO_2_ was significantly increased with all compression sleeves (*p* < 0.05) compared with no compression (from +6.9% for the least effective to +22.6% for the most effective). Large differences were observed between compression sleeves (*p* < 0.05). StO_2_ was positively correlated with compression pressure (*p* < 0.05; *r* = 0.84). *Conclusion*. This study shows that wearing compression sleeves from various brands differently affects tissue oxygen saturation. Differences were linked to the compression pressure: higher compression pressures were associated with higher StO_2_.

## 1. Introduction

Compression therapy is used for the treatment of venous pathologies such as deep vein thrombosis and chronic-venous insufficiency [1]. The application of the external pressure on the lower limbs acts to compress the veins thus reducing their diameter [2]. In consequence the velocity increases [2], which in turn encourages the return of blood to the heart and reduces pooling [2]. Many studies have demonstrated an increase in mean deep venous velocity, reduced venous pooling, and an improved venous return in patients who wore graduated compression stockings [3–5]. The use of compression garments in sport is becoming increasingly popular due to claims that they can improve recovery from exercise [6], by exerting these hemodynamic effects.

Evidence for the efficacy of compression garments in recovery is solid with recent meta-analysis supporting the use of compression to alleviate symptoms associated with fatigue [6, 7]. Suggested mechanisms include enhanced venous return and blood flow in passive conditions [8, 9] that may aid the removal of metabolic waste [10, 11], reduce edema [12, 13], and improve muscle oxygenation [14, 15]. However in some cases there is little evidence to support some of the purported benefits and gaps in knowledge are still evident [16, 17]. The heterogeneity of effects could be explained by the disparity in terms of compression garments in all studies [6, 7]. A consensus regarding the appropriate recommendations is also missing.

For example, findings of French et al. [16] indicate that recovery and regeneration appear unaffected by wearing compression garments for 12 h after a hard training session compared with passive recovery. However, it should be clearly stated that one limitation of this study is that compression garments used were reported to have an average compression pressure of 12 mmHg at the calf [16]. Indeed venous return and other hemodynamic benefits which may aid recovery were previously described to be significantly improved with a minimum compression pressure of 15–20 mmHg and with a peak for a pressure of around 40 mmHg [2, 18].

Some other studies present such limitation [19, 20] and recent meta-analysis suggest understanding the discrepancy of results well to focus on the characteristics of compression garments especially compression pressure [6, 7]. Therefore the aim of the present study was to assess the changes in tissue oxygen saturation (StO_2_) in response to the application of the main commercially available calf compression sleeves. We hypothesized that results may be strongly correlated with the compression pressure. We focused on StO_2_ because it has been extensively documented as a hemodynamic benefit of compression garments during recovery. It also presents the advantage of being noninvasively measured using near-infrared spectroscopy (NIRS) [21].

## 2. Materials and Methods

### 2.1. Participants

Eight healthy subjects (no history of cardiopulmonary disease or medication) were studied (mean ± SD: age, 32.4 ± 4.8 years; height, 174.5 ± 1.9 cm; body mass, 66.1 ± 2.2 kg; calf circumference at larger part: 36.4 ± 0.2 cm). They were informed about the procedures and risks associated with participation in the study and all provided their written informed consent prior to participation. The study protocol was approved by the local ethics committee and was in accordance with the Declaration of Helsinki of the World Medical Association with regard to research conduct.

### 2.2. Study Design

**Figure 1:**
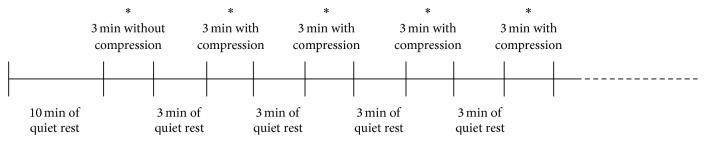
Experimental protocol. ^*∗*^StO_2_ measurements.

The subjects came to the laboratory (temperature: 20.0 ± 1.0°C; humidity: 50 ± 1.0%) to complete a session in seated position including 10 min of quiet rest followed by 3 min measuring calf StO_2_ without compression sleeves and then alternating of 3 min of quiet rest and 3 min measuring StO_2_ with calf compression sleeves. A total of 15 different commercially available compression sleeves were studied in a randomized order (Figure 1).

### 2.3. Measurements

Using NIRS technique, the InSpectra StO_2_ Tissue Oxygenation Monitor, Model 650 (Hutchinson, MN, USA), provides continuous noninvasive assessment of StO_2_ at a maximum depth of 15 mm. The measurement principles of this technology have been described [21] and its accuracy and reproducibility have been previously established [22]. The microcirculatory StO_2_ assessment is defined as the ratio [HbO_2_]/([Hb]+[HBO_2_]) expressed as percent, with HbO_2_ and Hb being oxy- and deoxygenated hemoglobin, respectively. The device does not display directly these values. StO_2_ was measured at the level of the right gastrocnemius muscle, 12 cm below the fibula head [14]. A transparent film was placed between the skin and the probe to protect it from sweat [23]. StO_2_ was measured during three minutes without compression and during three minutes with each calf compression sleeve. StO_2_ values were analyzed with StO_2_ Researcher's Analysis Software Version 4 (Hutchinson, MN, USA). Only the mean of the last minute of each 3 min period was considered.

### 2.4. Compression Sleeves

15 of main commercially available compression sleeves were studied (Table 1). Their size was chosen according to the individual calf circumference. The pressure of each calf sleeve was measured at the same area as the StO_2_ measurement using a pneumatic measuring system (Picopress, Microlabitalia, Padua, Italy) [24]. The pressure transducer consists of a flat plastic pressure probe (5 cm diameter) that is filled with 2 mL of air for the pressure measurement. Fluctuations of pressure on this probe are transformed into electronic signals that can be recorded continuously.

### 2.5. Statistical Analysis

Statistical analyses were performed using SigmaStat software for Windows 3.5 (Systat Software Inc., San Jose, CA, USA). Data are presented as mean ± SD. A *p* value <0.05 was considered as significant. The normality of distribution was tested using the Kolmogorov-Smirnov test. To assess the effects of the calf compression sleeves, StO_2_ was analyzed using Repeated Measures ANOVA on Ranks. Student-Newman-Keuls method was used for post hoc pairwise comparisons. Influence of compression pressure on StO_2_ was assessed using Pearson correlation.

## 3. Results

**Figure 2:**
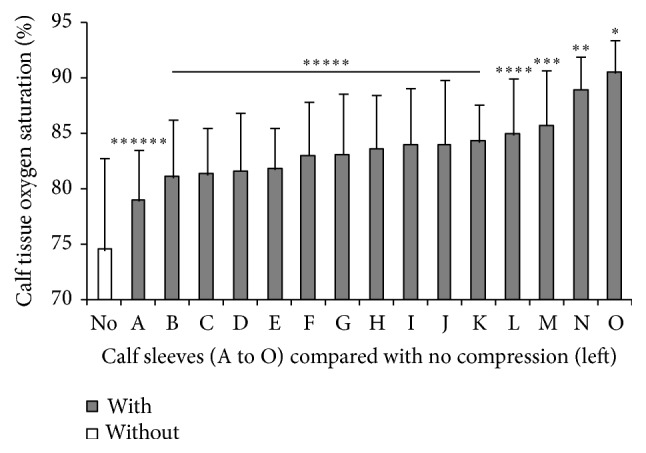
Calf StO_2_ recorded with or without calf compression sleeves. ^*∗*^Significantly different from all other calf sleeves including no compression. ^*∗∗*^Significantly different from A to M and no compression. ^*∗∗∗*^Significantly different from A to F and no compression. ^*∗∗∗∗*^Significantly different from A to E and no compression. ^*∗∗∗∗∗*^Significantly different from A and no compression. ^*∗∗∗∗∗∗*^Significantly different from no compression.

**Figure 3:**
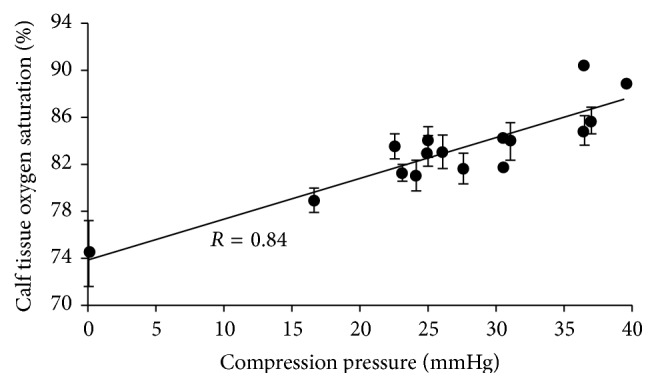
Influence of compression pressure (mmHg) on StO_2_.

StO_2_ recorded with or without compression sleeves is presented in Figure 2. StO_2_ was significantly increased with all compression sleeves (*p* < 0.05) compared with no compression (from +6.9% for A to +22.6% for O). Significant differences between compression sleeves were noted and are presented in Figure 3. Influence of compression pressure on StO_2_ is presented in Figure 3. StO_2_ was positively correlated with compression pressure (*p* < 0.05; *r* = 0.84).

## 4. Discussion

The present study aimed at investigating the changes in StO_2_ in response to the application of the main commercially available calf compression sleeves. Two major findings have been revealed. (1) Wearing all compression sleeves significantly increased StO_2_ and (2) the most effective increases of StO_2_ were correlated with the highest compression pressures. These results confirm our hypothesis and may be explained by several mechanisms.

### 4.1. Changes in StO_2_ with Calf Compression Sleeves

Firstly, StO_2_ increased with calf compression sleeves (from +6.9% for A to +22.6% for O). These results are in accordance with previous works that reported an increase in StO_2_ with calf compression sleeves before and after running [14] or cycling exercises [25]. This higher StO_2_ was attributed to the increased muscle flow rate [8, 9] and changes in skin blood flow [26, 27]. Indeed, wearing compression on the lower limbs is known to increase venous return [2], causing venous pressure to decrease [1]. Venous emptying may increase arteriovenous pressure gradient [28], increasing arterial flow rate, oxygen supply, and therefore StO_2_. The myogenic response may separately contribute to the higher StO_2_ [9]. As previously described [9], arterial vessels dilate in response to a fall of the transmural vessel pressure gradient. The pressure applied by sleeves is transmitted into the tissue and thus reduces the transmural pressure gradient of the arterial vessels [29]. Finally, changes in skin blood flow must also be considered [26, 27]. Indeed, StO_2_ was recorded at a maximum depth of 15 mm, including cutaneous and muscular vessels. Moreover, previous studies suggest that compression sleeves may affect cutaneous StO_2_ through temperature changes [14, 27] and pressure-induced skin vasodilation [26, 30].

### 4.2. Dose-Response: Higher Compression Pressures Were Associated with Higher StO_2_

This study also revealed that increase of calf StO_2_ with compression sleeves was positively correlated with compression pressure (*p* < 0.05; *r* = 0.84). This finding is in agreement with previous studies suggesting a dose-response of compression pressure on venous hemodynamics including the velocity of venous circulation, venous pump function, or the degree of decrease in edema [2, 3, 31]. However, the relation between the compression pressure and its effects on hemodynamics is not systematically demonstrated [31–33]. A possible explanation for this lack of relation is that hemodynamics may also be affected by other factors such as pressure gradient [24, 32] and elastic properties [34, 35]. For example, it has been shown that with the same pressure inelastic material is more effective than elastic [34, 35]. By extension the seamless knitting could be in favor of long lasting effects on StO_2_: no fraying and stitch defects making the sleeves loose fit and negatively affecting compression and elastic properties.

### 4.3. Practical Applications

This study provides support for the hypothesis that wearing compression sleeves from various brands may differently affect recovery, by virtue of their varied effects on StO_2_. These results contribute to understanding well the large differences reported in recent meta-analysis [6, 7] about effects of compression garments during recovery. This study also provides information about relation existing between compression pressure and calf StO_2_. Since higher compression pressure was associated with higher StO_2_ this work suggests focusing on compression garments with high pressure level for recovery. However further studies are requested to analyze the balance between effectiveness and comfort [2].

## 5. Conclusions

In conclusion, this study shows that wearing compression sleeves from various brands differently increases tissue oxygen saturation. Differences were linked to the compression pressure: higher compression pressures were associated with higher StO_2_.

## Figures and Tables

**Table 1:** Characteristics of calf compression sleeves.

Compression sleeves	Compression (mmHg)	Composition (%)			Knitting
		Polyamide	Elastane	Polyester	
A	16.5	45.0	7.0	48.0	Seamless
B	24.0	78.0	22.0	0	Seam
C	23.0	80.0	20.0	0	Seam
D	27.5	95.0	5.0	0	Seamless
E	30.5	77.0	23.0	0	Seamless
F	25.0	76.6	23.3	0.2	Seamless
G	26.0	74.0	26.0	0	Seamless
H	22.5	70.0	8.0	22.0	Seamless
I	25.0	90.0	10.0	0	Seamless
J	31.0	79.0	21.0	0	Seamless
K	30.5	79.0	21.0	0	Seamless
L	36.5	65.0	35.0	0	Seam
M	37.0	47.0	30	23	Seamless
N	39.5	60.0	25.0	15.0	Seamless
O	36.5	60.0	25.0	15.0	Seamless
